# The Covid19 outbreak: a catalyst for digitization in African countries

**DOI:** 10.1186/s42506-020-00047-w

**Published:** 2020-08-08

**Authors:** Said Bensbih, Hajar Essangri, Amine Souadka

**Affiliations:** 1 Laboratory of Mechanics, Production and Industrial Engineering (LMPGI), University Hassan II of Casablanca, ESTC, ENSEM, Casablanca, Morocco; 2 Surgical Oncology Department, National Institute of Oncology, University Mohammed V, Rabat, Morocco

To the Editor:

Digital health use in some African countries has prompted a significant transformation [1] and we would like to underline the Covid-19 outbreak’s role as a catalyst for digital health growth in Africa.

The Covid-19 outbreak is the global health crisis of the century with 210 affected countries and 10,599,620 million confirmed cases [2]. Alongside the human cost, the pandemic has taken a heavy toll on societies, international economy, and healthcare systems worldwide.

Containment measures were enforced to mitigate this impact, especially in countries with fragile health systems since slowing infection spread is the main method to tackle this crisis.

As schools and offices are closing, digitalization is key to survive. Remote work, E-learning, and the use of online services on an everyday basis are becoming the new norms, and digitization initiatives, such as the Morocco Digital Transformation Strategy [3] are fast tracked. Similarly, to reduce hospital-related transmission and infection, hospital organization and patient management are also shifting.

In medicine, the current situation revealed the possibility of harnessing the power of digital technology to fight the pandemic and benefit health systems in the long run [4]. Telemedicine is increasingly used with patients’ fear of contagion pushing them to avoid hospitals and as lockdown hinders normal consults. In Morocco, the ministry of health launched a free telemedicine platform to allow remote medical care in these times, and similar initiatives were adopted by a wide range of African countries. Technology is also implemented in appointment booking and triage of possible Covid-19 contacts.

On the other hand, with less than one physician per thousand people [5], doctor shortage in Africa is a challenge that technology could solve. Digital health will enable remote chronic disease follow-up, multidisciplinary video conference meetings, and specialized consults for hospitalized patients. This will compensate for the lack of resources and healthcare infrastructures alongside allowing for an even distribution of health services. In addition, as digital literacy is increased by the current circumstances, patients’ acceptance of remote health services will also change.

The current crisis, although putting strain on health systems worldwide, has allowed for the accelerated implementation of digital strategies in health services. This increased technology use can help breach healthcare inequities and be a step forward towards a more universal health coverage in an ever changing medicine.

## Data Availability

Not applicable
